# 2,2′-Dichloro-*N*,*N*′-[1,3-phenyl­enebis(methyl­ene)]diacetamide

**DOI:** 10.1107/S1600536812008653

**Published:** 2012-03-03

**Authors:** Hong-Xin Cai, Wei-Na Wu

**Affiliations:** a Department of Physics and Chemistry, Henan Polytechnic University, Jiaozuo 454000, People’s Republic of China

## Abstract

The complete mol­ecule of the title compound, C_12_H_14_Cl_2_N_2_O_2_, is generated by a crystallographic twofold axis with two C atoms of the central benzene ring lying on the axis. In the crystal, N—H⋯O hydrogen bonds link the mol­ecules into chains parallel to the *c* axis.

## Related literature

For the synthesis of lanthanide complexes with amide-type ligands, see: Wu *et al.* (2008 ▶). For a related structure, see: Yuan *et al.* (2010 ▶).
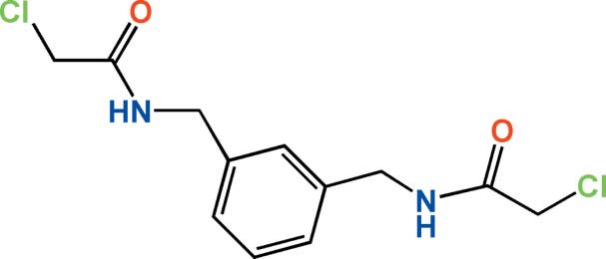


## Experimental

### 

#### Crystal data

C_12_H_14_Cl_2_N_2_O_2_
*M* *_r_* = 289.15
Monoclinic, 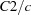

*a* = 20.62 (2) Å
*b* = 7.464 (8) Å
*c* = 9.485 (11) Å
β = 110.362 (11)°
*V* = 1369 (3) Å^3^
*Z* = 4
Mo *K*α radiation
μ = 0.47 mm^−1^
*T* = 296 K
0.27 × 0.23 × 0.22 mm

#### Data collection

Bruker SMART APEX CCD diffractometer
Absorption correction: multi-scan (*SADABS*; Bruker, 2007 ▶) *T*_min_ = 0.881, *T*_max_ = 0.902
6996 measured reflections
1574 independent reflections
1288 reflections with *I* > 2σ(*I*)
*R*_int_ = 0.080

#### Refinement

*R*[*F* ^2^ > 2σ(*F* ^2^)] = 0.055
*wR*(*F* ^2^) = 0.157
*S* = 1.06
1574 reflections
83 parameters
H-atom parameters constrained
Δρ_max_ = 0.46 e Å^−3^
Δρ_min_ = −0.31 e Å^−3^

### 

Data collection: *APEX2* (Bruker, 2007 ▶); cell refinement: *SAINT* (Bruker, 2007 ▶); data reduction: *SAINT*; program(s) used to solve structure: *SHELXS97* (Sheldrick, 2008 ▶); program(s) used to refine structure: *SHELXL97* (Sheldrick, 2008 ▶); molecular graphics: *SHELXTL* (Sheldrick, 2008 ▶); software used to prepare material for publication: *SHELXTL* ▶).

## Supplementary Material

Crystal structure: contains datablock(s) I, global. DOI: 10.1107/S1600536812008653/vm2160sup1.cif

Structure factors: contains datablock(s) I. DOI: 10.1107/S1600536812008653/vm2160Isup2.hkl

Supplementary material file. DOI: 10.1107/S1600536812008653/vm2160Isup3.cml

Additional supplementary materials:  crystallographic information; 3D view; checkCIF report

## Figures and Tables

**Table 1:** Hydrogen-bond geometry (Å, °)

*D*—H⋯*A*	*D*—H	H⋯*A*	*D*⋯*A*	*D*—H⋯*A*
N1—H1*C*⋯O1^i^	0.86	2.03	2.864 (3)	163

Symmetry code: (i) 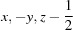.

**Fig. 1.**
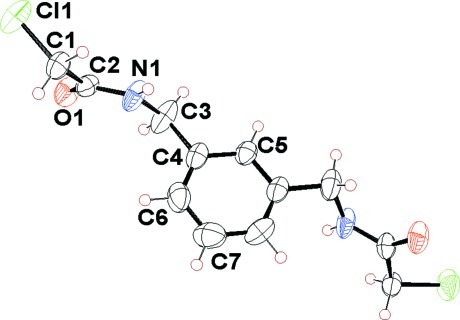
The molecular structure shown with 50% probability displacement ellipsoids. Unlabelled atoms are related with the labelled ones by symmetry operation (-x, y, -z - 1/2).

## supplementary crystallographic information

### Comment

The luminescent properties of the lanthanide complexes with amide type ligands have been investigated in our previous work (Wu *et al.*, 2008). As part of our ongoing studies of the amide type ligands, the title compound was synthesized and characterized by X-ray diffraction.

The complete molecule of the title compound (Fig. 1) is generated by a crystallographic twofold axis with atoms C5 and C7 of the central phenyl group lying on the axis. All the bond lengths are comparable with those observed in a similar compound (Yuan *et al.*, 2010). In the crystal, intermolecular N—H···O hydrogen bonds link the molecules into chains parallel to the *c* axis (Table 1).

### Experimental

A chloroform solution containing chloroacetyl chloride (2.26 g, 0.02 mol) was added dropwise to a solution of (3-(aminomethyl)phenyl)methanamine (1.36 g, 0.01 mol) and pyridine (1.60 g, 0.02 mol) in chloroform (20 ml) under stirring on a ice-water bath. Then, the reaction mixture was stirred at room temperature for 3.5 h. A solid product was separated from the solution by suction filtration, purified by washing with water, 0.5 mol/*L* HCl, 0.5 mol/*L* NaOH and distilled water, respectively. Colourless prism crystals were obtained by slow evaporation of the acetone solution at room temperature.

### Refinement

The H atoms were placed at calculated positions and refined in riding mode, with the carrier atom-H distances = 0.93 Å for aryl, 0.97 Å for methylene and 0.86 Å for the secondary amine H atoms. The *U*_iso_ values were constrained to be 1.2*U*_eq_ of the carrier atom for the H atoms.

### Crystal data

**Table:**

C_12_H_14_Cl_2_N_2_O_2_	*F*(000) = 600
*M**_r_* = 289.15	*D*_x_ = 1.403 Mg m^−^^3^
Monoclinic, *C*2/*c*	Mo *K*α radiation, λ = 0.71073 Å
Hall symbol: -C 2yc	Cell parameters from 1673 reflections
*a* = 20.62 (2) Å	θ = 2.1–27.5°
*b* = 7.464 (8) Å	µ = 0.47 mm^−^^1^
*c* = 9.485 (11) Å	*T* = 296 K
β = 110.362 (11)°	Prism, colorless
*V* = 1369 (3) Å^3^	0.27 × 0.23 × 0.22 mm
*Z* = 4	

### Data collection

**Table:**

Bruker SMART APEX CCD diffractometer	1574 independent reflections
Radiation source: fine-focus sealed tube	1288 reflections with *I* > 2σ(*I*)
Graphite monochromator	*R*_int_ = 0.080
φ and ω scans	θ_max_ = 27.5°, θ_min_ = 2.9°
Absorption correction: multi-scan (*SADABS*; Bruker, 2007)	*h* = −26→26
*T*_min_ = 0.881, *T*_max_ = 0.902	*k* = −9→9
6996 measured reflections	*l* = −12→12

### Refinement

**Table:**

Refinement on *F*^2^	Primary atom site location: structure-invariant direct methods
Least-squares matrix: full	Secondary atom site location: difference Fourier map
*R*[*F*^2^ > 2σ(*F*^2^)] = 0.055	Hydrogen site location: inferred from neighbouring sites
*wR*(*F*^2^) = 0.157	H-atom parameters constrained
*S* = 1.06	*w* = 1/[σ^2^(*F*_o_^2^) + (0.0746*P*)^2^ + 0.9706*P*] where *P* = (*F*_o_^2^ + 2*F*_c_^2^)/3
1574 reflections	(Δ/σ)_max_ < 0.001
83 parameters	Δρ_max_ = 0.46 e Å^−^^3^
0 restraints	Δρ_min_ = −0.31 e Å^−^^3^

### Special details

**Table:**

Geometry. All e.s.d.'s (except the e.s.d. in the dihedral angle between two l.s. planes) are estimated using the full covariance matrix. The cell e.s.d.'s are taken into account individually in the estimation of e.s.d.'s in distances, angles and torsion angles; correlations between e.s.d.'s in cell parameters are only used when they are defined by crystal symmetry. An approximate (isotropic) treatment of cell e.s.d.'s is used for estimating e.s.d.'s involving l.s. planes.
Refinement. Refinement of *F*^2^ against ALL reflections. The weighted *R*-factor *wR* and goodness of fit *S* are based on *F*^2^, conventional *R*-factors *R* are based on *F*, with *F* set to zero for negative *F*^2^. The threshold expression of *F*^2^ > σ(*F*^2^) is used only for calculating *R*-factors(gt) *etc*. and is not relevant to the choice of reflections for refinement. *R*-factors based on *F*^2^ are statistically about twice as large as those based on *F*, and *R*- factors based on ALL data will be even larger.

### Fractional atomic coordinates and isotropic or equivalent isotropic displacement parameters (Å^2^)

**Table:**

	*x*	*y*	*z*	*U*_iso_*/*U*_eq_	
C1	0.28959 (11)	−0.0305 (3)	0.6316 (2)	0.0460 (5)	
H1A	0.2913	0.0011	0.5338	0.055*	
H1B	0.2936	−0.1597	0.6422	0.055*	
C2	0.22101 (10)	0.0300 (3)	0.6421 (2)	0.0399 (5)	
C3	0.10062 (11)	0.1072 (4)	0.4959 (3)	0.0635 (8)	
H3A	0.0938	0.2349	0.4780	0.076*	
H3B	0.0931	0.0795	0.5889	0.076*	
C4	0.04867 (10)	0.0058 (3)	0.3676 (2)	0.0443 (5)	
C5	0.0000	0.0960 (4)	0.2500	0.0429 (7)	
H5	0.0000	0.2206	0.2500	0.052*	
C6	0.04839 (12)	−0.1788 (4)	0.3656 (3)	0.0599 (7)	
H6	0.0810	−0.2418	0.4427	0.072*	
C7	0.0000	−0.2710 (5)	0.2500	0.0701 (11)	
H7	0.0000	−0.3956	0.2500	0.084*	
N1	0.17173 (9)	0.0598 (3)	0.51078 (19)	0.0526 (6)	
H1C	0.1819	0.0510	0.4305	0.063*	
O1	0.21270 (9)	0.0449 (3)	0.76351 (17)	0.0564 (5)	
Cl1	0.35925 (3)	0.07271 (11)	0.77441 (8)	0.0728 (3)	

### Atomic displacement parameters (Å^2^)

**Table:**

	*U*^11^	*U*^22^	*U*^33^	*U*^12^	*U*^13^	*U*^23^
C1	0.0388 (11)	0.0610 (13)	0.0346 (10)	−0.0002 (9)	0.0081 (8)	−0.0056 (9)
C2	0.0372 (10)	0.0537 (12)	0.0294 (9)	−0.0106 (8)	0.0123 (8)	−0.0037 (8)
C3	0.0341 (11)	0.113 (2)	0.0410 (11)	0.0022 (12)	0.0095 (9)	−0.0225 (13)
C4	0.0299 (9)	0.0698 (14)	0.0344 (10)	0.0022 (9)	0.0129 (8)	−0.0017 (9)
C5	0.0319 (14)	0.0550 (18)	0.0414 (14)	0.000	0.0120 (12)	0.000
C6	0.0376 (11)	0.0767 (18)	0.0620 (14)	0.0108 (11)	0.0133 (10)	0.0208 (12)
C7	0.0496 (19)	0.052 (2)	0.108 (3)	0.000	0.027 (2)	0.000
N1	0.0323 (9)	0.0990 (17)	0.0264 (8)	−0.0014 (9)	0.0101 (7)	−0.0088 (8)
O1	0.0523 (10)	0.0911 (14)	0.0281 (7)	−0.0061 (8)	0.0170 (7)	−0.0022 (7)
Cl1	0.0404 (4)	0.1002 (6)	0.0649 (5)	−0.0068 (3)	0.0021 (3)	−0.0226 (4)

### Geometric parameters (Å, º)

**Table:**

C1—C2	1.520 (3)	C4—C6	1.378 (4)
C1—Cl1	1.771 (2)	C4—C5	1.388 (3)
C1—H1A	0.9700	C5—C4^i^	1.388 (3)
C1—H1B	0.9700	C5—H5	0.9300
C2—O1	1.227 (3)	C6—C7	1.382 (3)
C2—N1	1.324 (3)	C6—H6	0.9300
C3—N1	1.467 (3)	C7—C6^i^	1.382 (3)
C3—C4	1.514 (3)	C7—H7	0.9300
C3—H3A	0.9700	N1—H1C	0.8600
C3—H3B	0.9700		
			
C2—C1—Cl1	110.26 (16)	C6—C4—C5	118.4 (2)
C2—C1—H1A	109.6	C6—C4—C3	120.6 (2)
Cl1—C1—H1A	109.6	C5—C4—C3	121.0 (2)
C2—C1—H1B	109.6	C4^i^—C5—C4	122.0 (3)
Cl1—C1—H1B	109.6	C4^i^—C5—H5	119.0
H1A—C1—H1B	108.1	C4—C5—H5	119.0
O1—C2—N1	123.7 (2)	C4—C6—C7	120.5 (2)
O1—C2—C1	121.7 (2)	C4—C6—H6	119.8
N1—C2—C1	114.53 (19)	C7—C6—H6	119.8
N1—C3—C4	111.2 (2)	C6—C7—C6^i^	120.3 (4)
N1—C3—H3A	109.4	C6—C7—H7	119.9
C4—C3—H3A	109.4	C6^i^—C7—H7	119.9
N1—C3—H3B	109.4	C2—N1—C3	123.07 (19)
C4—C3—H3B	109.4	C2—N1—H1C	118.5
H3A—C3—H3B	108.0	C3—N1—H1C	118.5
			
Cl1—C1—C2—O1	−41.4 (3)	C5—C4—C6—C7	−0.9 (3)
Cl1—C1—C2—N1	140.17 (19)	C3—C4—C6—C7	178.68 (18)
N1—C3—C4—C6	58.1 (3)	C4—C6—C7—C6^i^	0.47 (15)
N1—C3—C4—C5	−122.3 (2)	O1—C2—N1—C3	−2.6 (4)
C6—C4—C5—C4^i^	0.46 (14)	C1—C2—N1—C3	175.8 (2)
C3—C4—C5—C4^i^	−179.1 (2)	C4—C3—N1—C2	−137.0 (2)

Symmetry code: (i) −*x*, *y*, −*z*+1/2.

### Hydrogen-bond geometry (Å, º)

**Table:**

*D*—H···*A*	*D*—H	H···*A*	*D*···*A*	*D*—H···*A*
N1—H1*C*···O1^ii^	0.86	2.03	2.864 (3)	163

Symmetry code: (ii) *x*, −*y*, *z*−1/2.
